# Integrating Weighted Gene Co-Expression Network and Differential Expression Analyses to Unveil the Role of RNA m6A Methylation Regulators in Idiopathic Parkinson’s Disease in Latin America

**DOI:** 10.3390/life16040592

**Published:** 2026-04-01

**Authors:** Francisco Leiva, Luis Constandil, Pedro Chana-Cuevas, Rene L. Vidal, Bernardo Morales, Rodrigo Vidal

**Affiliations:** 1 Laboratory of Genomics, Molecular Ecology and Evolutionary Studies, Department of Biology, Faculty of Chemistry and Biology, University of Santiago of Chile, Santiago 9170022, Chile; 2 Laboratory of Neurobiology, Department of Biology, Faculty of Chemistry and Biology, University of Santiago of Chile, Santiago 9170022, Chile; luis.constandil@usach.cl; 3 Faculty of Medicine, University of Santiago of Chile, Santiago 9170022, Chile; pedro.chana@usach.cl; 4 Center for Integrative Biology, Universidad Mayor, Santiago 7510041, Chile; rene.vidal@umayor.cl; 5 Laboratory of Neuroscience, Department of Biology, Faculty of Chemistry and Biology, University of Santiago of Chile, Santiago 9170022, Chile; bernardo.morales@usach.cl

**Keywords:** m6A methylation, m6A-related genes, Parkinson’s disease

## Abstract

Idiopathic Parkinson’s disease (iPD) represents the most prevalent form of Parkinson’s disease; however, the molecular mechanisms underlying its development remain only partially understood. N6-methyladenosine (m6A), the most abundant internal RNA modification in eukaryotic mRNA, has emerged as a key regulator of gene expression and has been implicated in neurodegenerative disorders. In this study, we performed integrated differential expression, weighted gene co-expression network analysis (WGCNA), and differential co-expression (DECO) analyses using peripheral blood RNA-seq data from Latin American controls and early iPD patients to investigate m6A-associated transcriptional alterations. WGCNA and differential expression analyses identified 1207 hub genes and 237 differentially expressed genes, respectively. The integration of these datasets with curated m6A-related genes yielded 12 overlapping candidate genes associated with early iPD. Subsequent DECO analysis revealed three significant m6A regulator–target differential co-expression links involving the m6A factors *VIRMA*, *YTHDF3*, and *HNRNPA2B1*. Experimental validation in an independent exploratory cohort confirmed altered expression of these regulators and increased m6A enrichment of *NRCAM* and *PKHD1* transcripts. To our knowledge, this study represents the first integrative transcriptomic evaluation of m6A-associated regulatory patterns in early iPD within a Latin American population. Collectively, our findings suggest that selective m6A-associated transcriptional network alterations may contribute to the systemic molecular signatures observed in early iPD, warranting further validation in larger and mechanistically oriented studies.

## 1. Introduction

Parkinson’s disease (PD) is the second most common age-related neurodegenerative disorder following Alzheimer’s disease, and affects more than 1% of the population aged 60 and older [1,2]. Globally, approximately 8.5 million individuals were living with PD in 2019, and the incidence of this disease is expected to double by 2040, affecting people of all ages, races, and ethnicities [3]. PD is characterized by the dysfunction and loss of dopamine-producing neurons in the substantia nigra (SN), a region of the midbrain. This neurodegeneration is caused by the accumulation of misfolded α-synuclein (α-syn), which aggregates into intracellular protein inclusions known as Lewy bodies. However, the molecular processes that govern α-syn fibrillization and the biogenesis of the LBs remain poorly understood [4,5]. In addition, only 15% of PD patients exhibit a family history of PD symptoms, with varying genetic predispositions, while the remaining 85% of the PD population are classified as iPD, which does not harbor an interpretable genetic cause [2,6,7]. Notably, recent reports suggest a higher prevalence of PD in Latin American populations compared with global estimates [8], highlighting the importance of investigating disease-associated mechanisms in underrepresented populations.

Epigenetic modifications play a crucial role in mediating environmental and/or genetic effects [9]. In this context, several studies have highlighted the importance of epigenetic modifications in the pathogenesis of PD [2,10]; however, these studies have focused mainly on the DNA level [11]. Epitranscriptomics (RNA modifications) has emerged as a new layer, bridging the gap between genomics vulnerabilities and the disease phenotype at the RNA level [12,13]. Although hundreds of RNA modifications have been described in coding and non-coding RNAs, the most prevalent and evolutionarily conserved eukaryotic RNA epimarker corresponds to N6-methyladenosine (m6A), which consists of a reversible addition of a methyl group (CH3) into the N6 nitrogen atom of an adenine. The regulation of m6A is dynamic and controlled by the machinery of RNA methyltransferases (writers, CH3 inclusion), m6A-binding proteins (readers, CH3 integration) and demethylases (erasers, CH3 removal) [14]. Today, it is widely accepted that m6A plays a key role in the regulation of several fundamental RNA processes, including nuclear export, degradation, translation, and splicing [14,15]. Increasing evidence suggests that m6A-mediated RNA regulation may influence biological processes directly implicated in PD pathogenesis, including neuronal survival, mitochondrial homeostasis, synaptic function, and neuroinflammatory responses [16,17,18,19]. However, despite these emerging links, only a limited number of studies have directly explored the role of m6A regulation in PD. For example, aberrant m6A levels have been reported in several brain regions—including the cerebellum, striatum, and SN—in PD mouse models and cellular systems, accompanied by transcriptional alterations in key components of the m6A regulatory machinery. These include upregulation of the m6A eraser *ALKBH5* together with downregulation of several other m6A regulatory factors such as *YTHDF1*, *METTL3*, and *HNRNPC* [20]. In addition, decreased m6A levels have been observed in peripheral blood mononuclear cells from PD patients in a Chinese cohort, where the m6A writer *METTL14* was proposed to regulate α-syn expression [20]. Collectively, these observations suggest that alterations in the transcriptional dynamics of m6A regulatory factors—including writers, erasers, and readers—may represent an important component of the molecular mechanisms contributing to PD pathogenesis.

Currently, the consolidation of robust bioinformatic approaches to analyze high-throughput measurements of gene expression information have led to important insights in the biomedical field of neurological diseases, including PD [21,22]. In particular, WGCNA, DECO and differential expression analyses are distinct strategies that can be utilized in a cooperative way to bridge the gap between individual m6A regulator genes and iPD phenotypes. Similar approaches have enabled the identification of seven key biomarker gene hub-modules associated with the diagnosis of iPD from blood tissue [23]. Latin American populations remain underrepresented in large-scale transcriptomic studies of Parkinson’s disease, as most available datasets derive from European or East Asian cohorts. Given the genetic admixture and environmental diversity characteristic of Latin American populations, evaluating transcriptomic alterations in this population may help determine whether previously reported molecular signatures are reproducible across diverse populations.

In this study, we obtained whole-blood transcriptome expression and clinical associated datasets of controls and iPD patients from the Accelerating Medicines Partnership-Parkinson’s Disease Initiative (AMP-PD) [24] to investigate m6A-associated transcriptional alterations in early iPD in a Latin American subpopulation cohort. By integrating WGCNA, differential expression, and DECO analyses, we aimed to identify candidate m6A-related regulatory interactions and generate hypotheses regarding systemic epitranscriptomic perturbations in early iPD in a Latin American cohort.

## 2. Materials and Methods

### 2.1. Data Acquisition and Preprocessing

**Figure 1:**
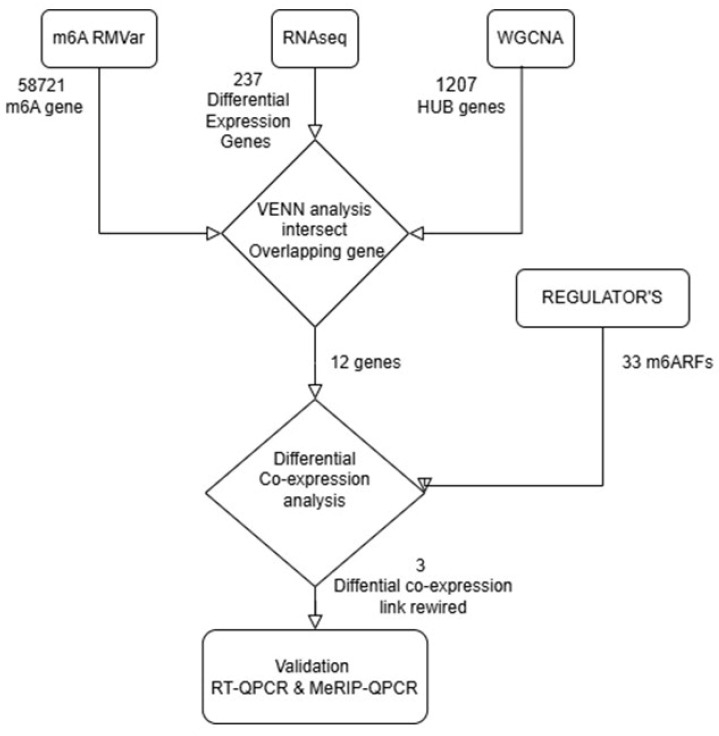
Integrative workflow of this study for identification of m6A-associated differential co-expression interactions in early idiopathic Parkinson’s disease (iPD). Schematic overview of the analytical pipeline used in this study. A total of 58,721 m6A-related genes were initially compiled from public databases (m6A RMVar). RNA-seq differential expression analysis identified 237 significantly differentially expressed genes (DEGs), while weighted gene co-expression network analysis (WGCNA) identified 1,207 hub genes associated with iPD. The intersection of m6A-related genes, DEGs, and WGCNA hub genes yielded 12 overlapping candidate genes. These candidates were subsequently evaluated using differential co-expression (DECO) analysis against a curated list of 33 m6A regulatory factors (m6ARFs). This analysis identified three significant m6A regulator–target differential co-expression links. Selected interactions were further assessed in an independent validation cohort using RT-qPCR and MeRIP-qPCR to evaluate gene expression and m6A methylation levels, respectively.

The workflow of this study is delineated in Figure 1. All the data of this research come from whole-blood RNA sequencing from the AMP-PD program [24], which integrates several programs/studies, including the Michael J. Fox Foundation for Parkinson’s Research (MJFF) and National Institutes of Neurological Disorders and Stroke (NINDS) BioFIND study, the Harvard Biomarkers Study (HBS) and the Stephen & Denise Adams Center for Parkinson’s Disease Research of Yale School of Medicine (CPDR-Y), the National Institute on Aging (NIA) International Lewy Body Dementia Genetics Consortium Genome Sequencing in Lewy Body Dementia Case-control Cohort (LBD), the MJFF LRRK2 Cohort Consortium (LCC), the NINDS Parkinson’s Disease Biomarkers Program (PDBP), the MJFF Parkinson’s Progression Markers Initiative (PPMI), the NINDS Study of Isradipine as a Disease-modifying Agent in Subjects With Early Parkinson Disease, Phase 3 (STEADY-PD3) and the NINDS Study of Urate Elevation in Parkinson’s Disease, Phase 3 (SURE-PD3). AMP-PD details about protocols procedures and data compilation can be found at https://www.amp-pd.org (accessed on 14 May 2025). We downloaded the total current AMP-PD dataset of 10,609 harmonized processed libraries (raw count matrices) corresponding to 3566 and 4376 iPD and control participants, respectively, and their associated clinical data. Distinct filters were applied to avoid potential biases. First, participants with available clinical and demographic metadata were selected based on the race and ethnicity fields provided in the AMP-PD dataset and diagnostic classification. Specifically, individuals classified as “White” in the Race metadata field and “Latino” in the Ethnicity metadata field, and assigned as confirmed iPD cases in the AMP-PD diagnostic metadata, were included in the analysis. Participants from other racial or ethnic backgrounds or with different conditions were not included. Restricting the analysis to participants self-identifying as “white” and “Latin” was implemented to reduce potential population stratification effects and to focus on a relatively homogeneous Latin American subgroup. The AMP-PD participant dataset cohort was structured in seven-point visits (months 0, 0.5, 6, 12, 18, 24 and 30+). As the focus of our study was the early iPD stage, only the samples of participants (controls and patients) with point visits at 0 and 0.5 months, and without diagnostic conflict along the rest of point visits, were selected. Then, participant samples with quality libraries (RIN) > 8 and plates with ≥3 libraries were kept for the next analysis. The HTSFilter package v. 1.49.0 [25] was utilized to filter low gene count reads and the PcaGrid function, from the rrcov R package v. 1.7-7 [26], to detect outliers. After filter steps and sequence quality control settings, a Latin American dataset of 23,559 genes from 44 samples (26 IPDs patients and 18 controls) was built (Supplementary Table S1; Supplementary Figures S1 and S2).

### 2.2. Differential Gene Expression Analysis

To minimize potential confounding effects in peripheral blood transcriptomic analyses, we systematically evaluated latent sources of variation and immune cell composition prior to differential expression modeling. Surrogate variable analysis (SVA) was performed using the sva R package v. 3.52.0 (https://bioconductor.org/packages/sva, accessed on 1 October 2025) to identify hidden sources of variation not captured by recorded clinical metadata. The estimated surrogate variables (SVs) were examined for biological relevance by calculating Pearson correlation coefficients against available clinical laboratory parameters, including absolute leukocyte counts (neutrophils, monocytes, and other subsets), as well as RIN, age and sex. In parallel, immune cell-type composition was estimated using CIBERSORTx (https://cibersortx.stanford.edu/, accessed on 5 October 2025) with the LM22 leukocyte gene signature matrix. Deconvolution was performed in relative mode using 1000 permutations to estimate statistical confidence in the inferred cell fractions. Batch correction within CIBERSORTx was not applied because the RNA-seq data were obtained from a harmonized AMP-PD processing pipeline. The resulting estimates represent relative immune cell proportions per sample that sum to one. These proportions were compared between iPD and control groups and evaluated as potential covariates in downstream analyses. Age and sex were the only variables significantly contributing to transcriptomic variance structure (Supplementary Figure S3). Differential expression analysis (Wald test) among iPD patients and controls was performed using the DESeq2 package v.1.46.0 [27] on the raw count matrix obtained after quality control and filtering. Sex and age were included as covariates. Genes with a log2Fold Change (FC) > |1.0| and an adjusted *p*-value of <0.05 were considered differentially expressed (DEGs). The packages in R, EnhancedVolcano v. 1.27.0 [28] and Pheatmap v.1.0.13 [29] were used to generate volcano plots and heatmaps (using VST-transformed counts from DESeq2 and row-scaled, Z-scores), respectively.

To gain insight into DEG-associated pathways and biological functions, KEGG pathways and Gene Ontology (GO) enrichment analyses were performed with the Cluster Profiler v. 4.4.4 package [30]. Over-representation analysis was conducted using a hypergeometric test to assess whether specific GO terms or KEGG pathways were significantly enriched among the selected gene sets compared to the background gene universe. The background set was defined as all genes retained after quality control and expression filtering. To account for multiple testing, *p*-values were adjusted using the Benjamini–Hochberg false discovery rate (FDR) correction method. Pathways and GO terms with adjusted *p*-values < 0.05 and fold enrichments > 1.5 were considered enriched. Correction was applied independently within each annotation category (e.g., GO Biological Process, KEGG pathways).

### 2.3. Gene Co-Expression Network and Hub Gene Identification

We used the WGCNA package v. 1.73 [31] to construct weighted gene co-expression networks from the normalized variance-stabilized (VST) expression matrix. A signed network was constructed to preserve the directionality of gene–gene correlations. The soft-thresholding power (β) was selected using the scale-free topology criterion via the pickSoftThreshold function. The optimal power (β = 16) was chosen as the lowest value, achieving a scale-free topology fit index > 0.85 while maintaining adequate mean connectivity. Modules were identified using hierarchical clustering of the topological overlap matrix (TOM) followed by dynamic tree cutting (minModuleSize = 30; mergeCutHeight = 0.1; maxBlockSize = 5000). Module eigengenes (MEs) were defined as the first principal component of each module. Gene significance (GS) and module membership (MM) were calculated using Pearson correlations between gene expression and iPD status or module eigengenes, respectively. Genes with GS > 0.2 and MM > 0.8 were defined as hub genes. Given the limited sample size, formal module preservation statistics based on independent cohorts were not feasible. Instead, network robustness was assessed through sensitivity analyses by evaluating alternative soft-thresholding powers (β = 14–18) while maintaining constant module detection parameters. Module–trait associations and hub gene identification were examined for stability across parameter settings, and key modules demonstrated consistent eigengene–trait relationships and substantial hub gene retention. Previous methodological studies have shown that WGCNA networks can be reliably constructed with sample sizes above approximately 20 samples, although larger cohorts improve network stability and reproducibility. In this context, the present dataset exceeds the recommended minimum sample size for exploratory network construction.

### 2.4. m6A-Related Genes and Overlapping Gene Analysis

A total of 17,742 human high-confidence (level 1) m6A-related genes were downloaded from the RMVAR v. 2.0 database [32]. The m6A-related gene set was intersected with the DEG and hub gene sets previously obtained in this study, to generate overlapping (common) genes among the three gene sets.

### 2.5. Differential Co-Expression Among m6A RNA Methylation Regulatory Factors and Overlapping Genes

Thirty-three common m6A RNA methylation regulatory factors (m6ARFs) were compiled from the literature (Supplementary Table S2). To assess the regulatory relationships between the compiled m6ARFs and overlapping genes, a differential co-expression (DECO) gene network analysis was performed using the diffcoexp v.1.28.0 package [33] with default parameters. This package addresses the gene regulatory relationships among two conditions (e.g., control and iPD patients) in terms of Differential Co-expressed Links (DCLs) and Differentially Co-expressed Genes (DCGs). DCLs correspond to gene pairs with statistically different correlation coefficients under two conditions, while DCGs refer to genes with significantly greater DCLs than expected at random. The RNA count data were normalized, and homoscedasticity was ensured using the VST from the DESeq2 framework. For each gene pair, the Pearson correlation coefficients (r1 and r2) were calculated separately for each group. To facilitate the formal comparison of these correlations, Fisher’s Z-transformation was applied to convert the correlation coefficients into Z-scores, which follow a normal distribution. The significance of the difference between the two Z-scores was evaluated using a Z-test, and the resulting *p*-values (1000 permutations) were adjusted for multiple testing using the Benjamini–Hochberg false discovery rate (FDR) method. Differentially co-expressed links (DCLs) were defined as gene pairs exhibiting statistically significant correlation differences (FDR < 0.05) and absolute correlation coefficients > 0.7 in at least one condition. The threshold of |r| > 0.7 was selected to prioritize strong correlation differences and to reduce the likelihood of detecting weak or unstable associations in the context of a moderate-sized transcriptomic dataset. In total, pairwise correlations were evaluated between 33 m6A regulatory factors and 12 overlapping candidate genes, resulting in 396 regulator–target gene pairs tested. Multiple testing correction using the FDR method was applied across all tested gene pairs. To evaluate the robustness of the identified DCLs, sensitivity analyses were conducted by (i) relaxing the absolute correlation threshold to |r| > 0.6, and (ii) assessing the stability of the direction and magnitude of correlation change (Δr) between conditions. The significant m6A regulator–target interactions identified under the primary threshold remained statistically significant and preserved their directionality under these alternative parameter settings, indicating robustness of the detected differential co-expression relationships.

### 2.6. Ethics Statement

This study was conducted according to the principles of the Declaration of Helsinki, and the protocol was approved by the Ethics Committee of Universidad de Santiago de Chile 752/2023. Written informed consent was obtained from all participants. Investigators were not blinded to disease conditions during experiments and setup of the results.

### 2.7. Sample Material and RNA Isolation

Peripheral venous blood samples were collected from 10 early iPD patients (diagnosed < 15 months and without causative variants in leucine-rich repeat kinase 2, *LRRK2*; glucosidase beta acid 1, *GBA*; synuclein alpha, *SNCA*; PTEN-induced kinase 1, *PINK1*; and parkin RBR E3 ubiquitin protein ligase, *PRKN*) and 10 sex-matched healthy volunteers, who served as non-randomized control subjects, enrolled in the Movement Disorder Center in Santiago, Chile (CETRAM; https://cetram.cl/, accessed on 14 May 2025) (Supplementary Table S3). No formal power calculation was performed prior to recruitment of the validation cohort, as this component of the study was designed as an exploratory, hypothesis-supportive analysis following transcriptomic discovery. The recruitment was conducted at a specialized movement disorder center and focused on early, clinically well-characterized iPD patients without known pathogenic variants, which inherently limits rapid large-scale enrollment. Although the resulting sample size (*n* = 10 per group) is modest, it reflects a carefully phenotyped clinical cohort and provides independent biological support for the transcriptomic observations. Total RNA was extracted from blood samples using the DNeasy Blood & Tissue Kit (Qiagen, Hilden, Germany), following the manufacturer’s protocol to ensure high-quality RNA recovery. RNA integrity was assessed by agarose gel electrophoresis, while concentration was measured using a NanoDrop One (Thermo Fisher Scientific, Wilmington, DE, USA) equipment.

### 2.8. Quantitative Reverse Transcription PCR (RT-qPCR) of m6A Regulator Factors

Three m6A regulator factors, *VIRMA* (vir-like m6A methyltransferase), *HNRNPA2B1* (heterogeneous nuclear ribonucleoprotein A2/B1) and *YTHDF3* (YTH N6-methyladenosine RNA binding protein F3) were selected to evaluate blood expression levels in iPD samples and control subjects. The total RNA previously isolated was used to synthesize cDNA using the Maxima First Strand cDNA Synthesis Kit (Thermo Fisher Scientific, Wilmington, DE, USA) followed by quantitative PCR using gene-specific primers (Supplementary Table S4). The *PSMD6* (proteasome 26S subunit, non-ATPase 6) gene was employed as the internal reference gene to normalize gene expression levels [34]. Reactions were prepared with FastStart SYBR Green Master Mix (Roche Diagnostics, Mannheim, Germany) and run in triplicate on the Rotor-Gene Q real-time PCR cycler (Qiagen, Hilden, Germany) machine, according to the manufacturer’s instructions. Thermal cycling conditions included an initial denaturation at 95 °C for 10 min, followed by 40 cycles at 95 °C for 15 s at 60 °C for 1 min. The relative expression of m6A regulator factors was estimated using the ΔΔCt method [35].

### 2.9. MeRIP-qPCR Analysis

MeRIP-qPCR was performed to quantify the m6A modification levels of two overlapping genes, namely *NRCAM* (neuronal cell adhesion molecule) and *PKHD1* (polycystic kidney and hepatic disease 1). The initial immunoprecipitation step was conducted using the EpiMark N6-Methyladenosine Enrichment Kit (New England Biolabs, Ipswich, MA, USA), according to the manufacturer’s instructions. Briefly, fragmented RNA (~200 nucleotides) was incubated with an anti-m6A antibody bound to Protein G Magnetic Beads (New England Biolabs, S1430) in 1× reaction buffer (150 mM NaCl, 10 mM Tris-HCl, pH 7.5, 0.1% NP-40 in nuclease-free water). The bead–antibody–RNA complex was supplemented with RNase inhibitor and incubated overnight at 4 °C with gentle rotation. After washing, m6A-enriched RNA was eluted and purified, then reverse-transcribed into cDNA using the protocol described above. In meRIP-qPCR, the unit of measurement for each sample analyzed is the percentage of IP/input ratio calculated according to 2 − ΔCt (ΔCt =  CtIP − Ctinput) and the ratio of IP RNA template and input RNA template to the initial RNA used for reverse transcription. Gene-specific primers are detailed in Supplementary Table S4.

### 2.10. Receiver Operating Characteristic Analysis

Receiver operating characteristic (ROC) curve analysis was performed to evaluate the discriminative capacity of candidate genes identified from the differential co-expression network. Gene expression values were used as continuous predictors, and disease status was defined as a binary outcome variable. For individual gene evaluation, ROC curves were generated using the pROC package in R (https://cran.r-project.org/web/packages/pROC/index.html, accessed on 14 September 2025). The area under the curve (AUC) was calculated to quantify diagnostic performance. Ninety-five percent confidence intervals (95% CI) were estimated using bootstrap resampling. To assess the combined predictive performance, a multivariable logistic regression model was constructed including *HTR4*, *NRCAM*, and *KIR3DL1* as independent variables. Predicted probabilities derived from the fitted model were used to generate the ROC curve for the combined classifier. The AUC and its corresponding 95% CI were computed to compare the discriminative accuracy between individual genes and the combined model. A two-sided *p* value < 0.05 was considered statistically significant.

## 3. Results

### 3.1. Differential Gene Expression Landscape

**Figure 2:**
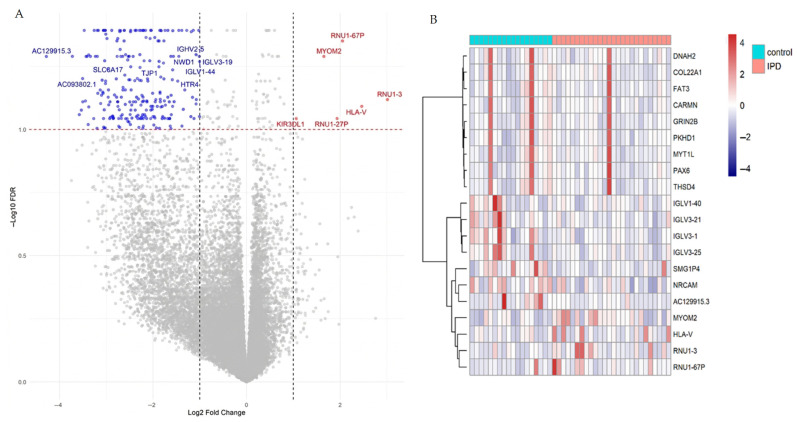
DEGs analysis on idiopathic Parkinson’s disease patients. (**A**) Volcano plot of gene expression of 44 AMP early/control Latin American iPD datasets, displaying log2 fold change (x-axis) versus -log10 FDR (y-axis) for gene expression data. Significant genes (*p*-value < 0.05 and |log2 (Fold Change)| > 1.0) are marked in blue (downregulated, left) and red (upregulated, right), with gene labels colored accordingly. Threshold lines are visible for statistical significance (horizontal, red) and fold change cutoffs (vertical, black). (**B**) Heat map of the DEGs, showing the 20 TOP differentially expressed genes ranked by adjusted *p*-value < 0.05 and row-scaled expression. Red represents higher expression, and blue represents lower expression. Analyses were performed using the AMP-PD discovery cohort (*n* = 44 participants; 26 iPD patients and 18 controls), with one RNA-seq library per individual included after quality filtering.

To identify the significantly affected iPD genes, a differential expression analysis was performed among controls and iPD patients. A total of 237 DEGs (|log2FC| ≥ 1.0 and adjusted *p*-value < 0.05) were detected, with a significant increase in gene downregulation in iPD patients, with 231 (97.47%) DEGs exhibiting downregulation and 6 (2.53%) upregulation (Figure 2A) (Supplementary Table S5). A heatmap analysis revealed distinct expression patterns among iPD patients and control subjects (Figure 2B), based on the top differentially expressed genes.

**Figure 3:**
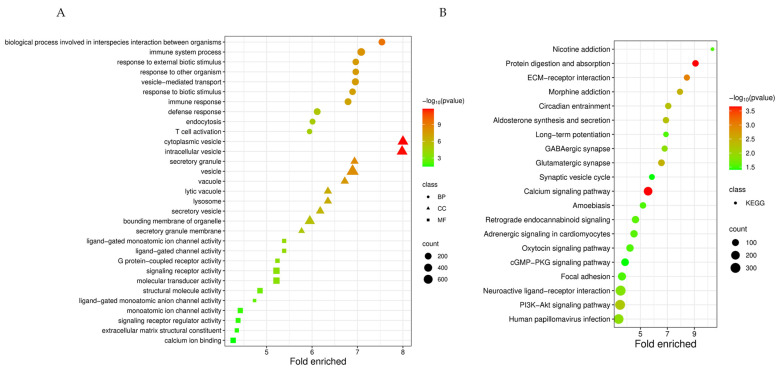
Functional enrichment analysis of differentially expressed genes (adjusted *p*-values < 0.05 and fold enrichments > 1.5) identified in peripheral blood from early iPD patients. (**A**) GO enrichment analysis, significantly enriched biological process (BP), cellular component (CC), and molecular function (MF) categories are shown. Symbol shapes denote GO classes, as indicated in the legend: circles represent BP, triangles rep-resent CC, and squares represent MF. (**B**) KEGG pathway enrichment analysis of the same gene set. The x-axis represents fold enrichment, dot size corresponds to gene count per term, and color scale indicates statistical significance (−log10-adjusted *p*-value). Enrichment analyses were performed using multiple testing correction, and only significantly enriched terms and pathways (adjusted *p*-values < 0.05 and fold enrichments > 1.5) are displayed. Analyses were performed using the AMP-PD discovery cohort (*n* = 44 participants; 26 iPD patients and 18 controls), with one RNA-seq library per individual included after quality filtering.

KEGG pathways and GO functional enrichment were performed to evaluate the potential functions of the DEGs detected. The significantly enriched GO biological terms principally included terms associated with the immune response (e.g., immune system processes: GO:0045087, defense response: GO:0006952 and T-cell activation: GO:0042110), cellular processes (e.g., endocytosis: GO:0006897) and response to stimulus (e.g., response to biotic stimulus: GO:0009607) (Figure 3A) (Supplementary Table S6). The KEGG analysis showed 20 significantly enriched pathways, with the top five corresponding to protein digestion and absorption (PATH: hsa04974), ECM–receptor interaction (PATH: hsa04512), morphine addiction (PATH: hsa05032), circadian entrainment (PATH: hsa04713) and aldosterone synthesis secretion (PATH: hsa04925) (Figure 3B) (Supplementary Table S7). Morphine addiction and protein digestion likely reflect shared gene annotations rather than disease-specific mechanisms, and should therefore be interpreted cautiously.

### 3.2. WGCNA and iPD Hub Gene Identification

**Figure 4:**
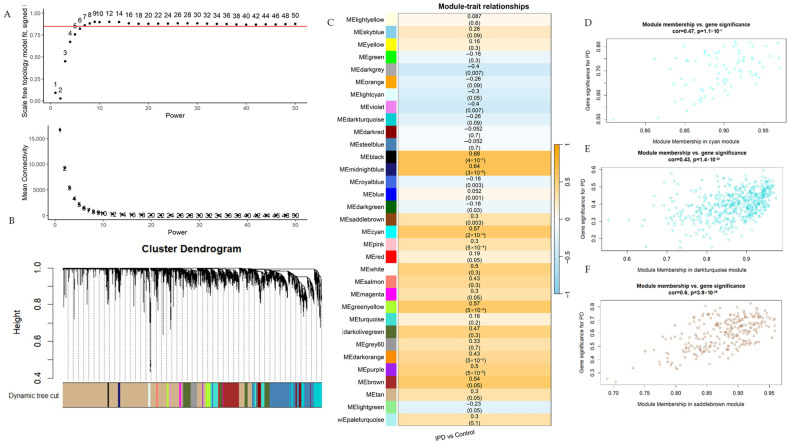
Early iPD-associated modules identified using weighted gene co-expression network analysis. (**A**) Scale-free topology analysis for soft-thresholding power selection. The upper panel shows the scale-free topology fit index (R^2^) as a function of the soft-thresholding power (β), with the selected power indicated at the point achieving R^2^ > 0.85 while maintaining adequate mean connectivity (lower panel). (**B**) Hierarchical clustering dendrogram of genes based on topological overlap, with modules identified using dynamic tree cutting and represented by distinct colors. (**C**) Module–trait relationship heatmap displaying Pearson correlation coefficients between module eigengenes and iPD status. Color intensity reflects the strength and direction of association, and corresponding *p*-values are indicated within each cell. (**D**–**F**) Trait–module relationships and feature modules of GS and MM, with each point defining a specific gene within every module that was plotted on the y-axis and the x-axis by GS and MM, respectively. (**D**–**F**) Scatterplots illustrating the relationship between module membership (MM) and gene significance (GS) for representative iPD-associated modules. Each point represents an individual gene within the module. Network construction and module–trait analyses were performed using the AMP-PD discovery cohort (*n* = 44 participants; 26 iPD patients and 18 controls), with one RNA-seq library per individual included after quality filtering.

To better understand the potential iPD genes interactions, a WGCNA was conducted for 23,559 genes from the Latin American iPD patient-control dataset. The scale-free fit indices at various soft-threshold values and their respective average connectivity are shown in Figure 4A. Figure 4B showcases the module clustering tree through the dynamic tree clipping option. A total of 33 co-expressed gene modules were generated for all the samples. Sensitivity analyses using alternative soft-thresholding powers (β = 14–18) demonstrated preservation of the main module structure and directional consistency of module–trait correlations. The majority of hub genes identified under β = 16 were retained across adjacent parameter settings, supporting the stability of hub gene detection despite the limited sample size. The correlation analysis among the modules obtained and the iPD traits revealed seven correlation coefficients (r) > |0.6| and *p*-values < 0.05. The modules showing the strongest associations with iPD status included the cyan, pink, midnightblue, darkturquoise, purple, turquoise, and black modules (Figure 4C). MM (module membership < 0.8) and GS (gene significance > 0.2) analyses identified 1207 hub genes (Figure 4D–F) in these seven modules.

### 3.3. iPD m6A-Related Genes

**Figure 5:**
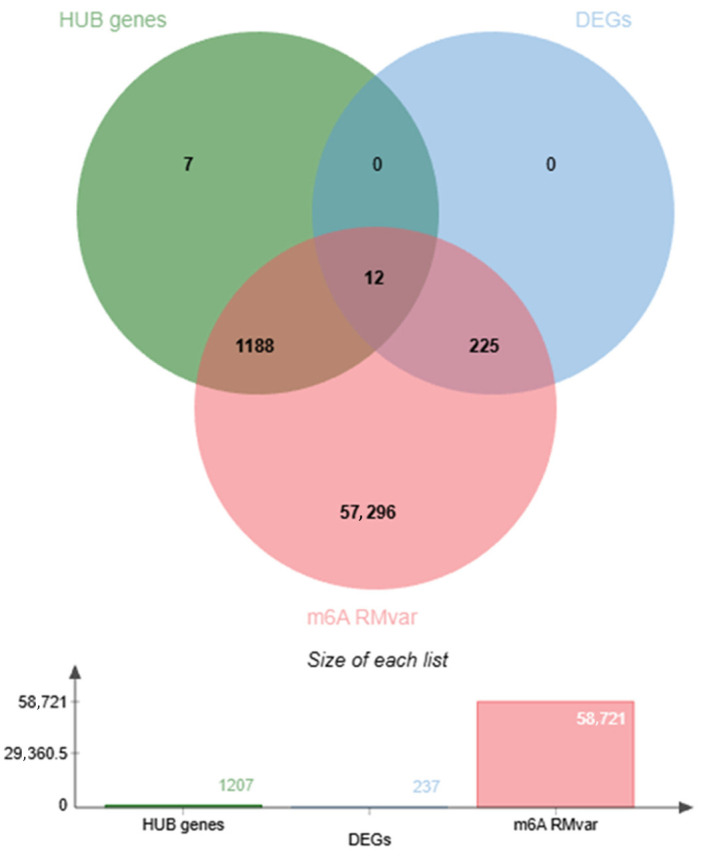
Integrative overlap analysis of m6A-related genes, differentially expressed genes, and WGCNA hub genes in early iPD. Venn diagram illustrating the intersection between WGCNA-identified hub genes (*n* = 1207), differentially expressed genes (DEGs; *n* = 237), and m6A-related genes compiled from the RMVar database (*n* = 58,721). Twelve genes were shared across all three datasets, representing candidate m6A-associated genes for downstream differential co-expression analysis. Numbers within each section indicate the count of genes unique or shared among categories. The bar plot below depicts the total size of each gene list prior to intersection. The gene sets used in this integrative analysis were derived from the AMP-PD discovery cohort (*n* = 44 participants; 26 iPD patients and 18 controls), with one RNA-seq library per individual included after quality filtering.

To determine potential iPD m6A-related genes, we retrieved 58,721 human m6A-related genes (level 1, including coding and noncoding genes) from RMVar 2.0, which were intersected with the sets of 12,107 hub genes and the 237 previously obtained DEGs. A total of twelve overlapping (common) iPD m6A-related genes were determined, including three upregulated (*RNU1-27P*: RNA U1 small nuclear 27, pseudogene; *KIR3DL1*: killer cell immunoglobulin-like receptor 3DL1 and *RNU1-67P*: RNA U1 small nuclear 67, pseudogene) and nine downregulated DEGs (*HTR4*: 5-hydroxytryptamine receptor; *NRCAM*: neuronal cell adhesion molecule; *MAGI1*: membrane-associated guanylate kinase, WW and PDZ domain-containing 1; *CFAP46*: cilia- and flagella-associated protein 46; *DNAH2*: dynein axonemal heavy chain 2, *PKHD1*: polycystic kidney and hepatic disease 1; *PTPN14*: phosphatase non-receptor type 14; *KIF26B*: kinesin family member 26B and *NWD1*: NACHT and WD repeat domain containing 1). These overlapping iPD m6A-related genes were found to be associated with several biological functions, including immune response-regulating signaling pathways, chemical synaptic transmission, cell–cell adhesion, signal transduction, cilium movement involved in cell motility, intracellular calcium ion homeostasis and lymphangiogenesis (Supplementary Table S8, Supplementary Figure S4 and Figure 5).

### 3.4. Differential Co-Expression Among m6ARFs and Overlapping Genes

To assess the regulatory relationships between m6ARFs (*n* = 33) and overlapping genes (*n* = 12) a differential co-expression analysis was performed using their links and correlations as the basis. The differential co-expression network revealed five hub genes and three significant rewiring co-expressed links, VIRMA–NRCAM (FDR = 0.0143), HNRNPA2B1–PKHD1 (FDR = 0.0143), and YTHDF3–NRCAM (FDR = 0.0317), all exhibiting strong correlation differences between conditions (|r| > 0.7) (Table 1). Sensitivity analyses confirmed that the three significant rewiring co-expressed links remained detectable after applying alternative correlation thresholds (|r| > 0.6) and assessing the stability of correlation change (Δr), supporting the robustness of these interactions.

### 3.5. Confirmation by RT-qPCR and MeRIP-qPCR

**Figure 6:**
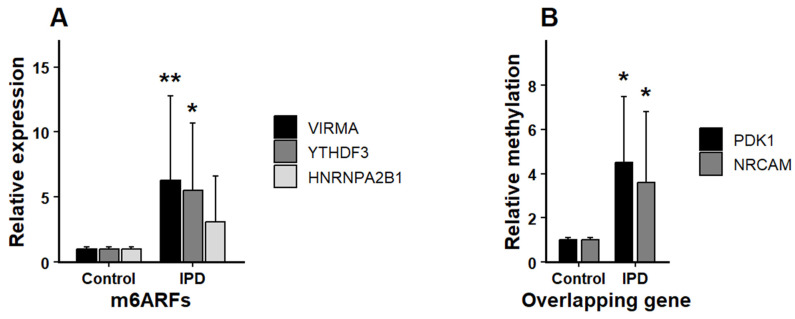
Experimental confirmation of m6A regulatory factors and target genes in an independent cohort. Bar plots illustrate the expression and methylation differences between control individuals and early iPD patients (*n* = 20; 10 controls and 10 early iPD). (**A**) Relative mRNA expression levels of the m6A regulatory factors *VIRMA*, *YTHDF3*, and *HNRNPA2B1*, quantified by RT-qPCR. Reactions were performed in technical triplicates for each sample, and expression levels were normalized to the reference gene using the 2ΔΔCt method. (**B**) Relative m6A methylation enrichment levels of *PDK1* and *NRCAM*, assessed by MeRIP-qPCR and expressed relative to input controls. Bars represent mean ± standard deviation (SD). Statistical comparisons between groups were performed using two-tailed Wilcoxon rank-sum tests. * *p* < 0.05; ** *p* < 0.01.

RT-qPCR was utilized in this study to assess the transcriptional levels of the three key drivers of regulatory m6ARFs network remodeling (*VIRMA*, *YTHDF3* and *HNRNPA2B1*) in blood tissue from Chilean iPD patients and control subjects. As shown in Figure 6A, the expression of *HNRNPA2B1* was significantly lower (*p* < 0.05) in iPD patients than in control subjects, while the expression of *VIRMA* and *YTHDF3* was significantly higher (*p* < 0.05). MeRIP-qPCR was used to assess the m6A methylation level of the two genes involved in the differential co-expression network modules (*NRCAM* and *PKHD1*) in both iPD patient and control groups. Both genes revealed significantly higher m6A methylation levels in iPD patients than in control subjects (Figure 6B).

### 3.6. Diagnostic Performance of Key Network Genes

**Figure 7:**
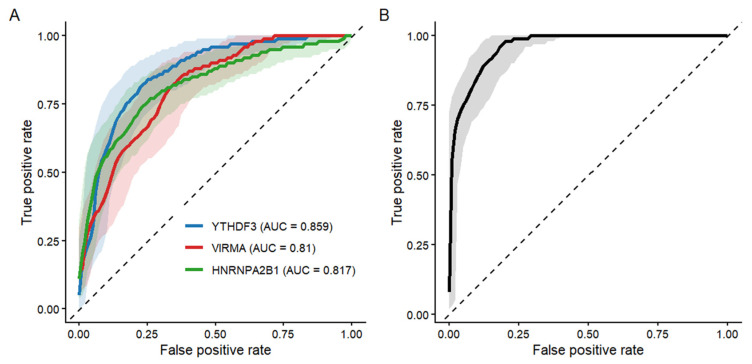
Receiver operating characteristic curve analysis of individual genes and combined model. (**A**) ROC curves illustrating the discriminative performance of *YTHDF3*, *VIRMA*, and *HNRNPA2B1* based on their expression profiles. Shaded areas represent 95% confidence intervals (CIs). The area under the curve (AUC) values were 0.859 for YTHDF3, 0.810 for *VIRMA*, and 0.817 for *HNRNPA2B1*. (**B**) ROC curve of the multivariable logistic regression model incorporating *YTHDF3*, *VIRMA*, and *HNRNPA2B1* expression levels. Shaded areas represent the 95% CI. ROC analyses were performed using the independent validation cohort (*n* = 20 participants; 10 early iPD patients and 10 controls).

To preliminarily assess the potential discriminative ability of candidate m6A regulatory factors identified from the differential co-expression network, ROC curve analysis was performed (Figure 7). Among the evaluated genes, *YTHDF3* showed the highest area under the curve (AUC = 0.859, 95% CI: 0.74–0.97), followed by *HNRNPA2B1* (AUC = 0.817, 95% CI ≈ 0.69–0.95) and *VIRMA* (AUC = 0.810, 95% CI: 0.68–0.94). These findings suggest a moderate-to-high capacity to distinguish cases from controls within the analyzed dataset. To further explore whether combining these markers could enhance classification performance, a multivariable logistic regression model incorporating *YTHDF3*, *VIRMA*, and *HNRNPA2B1* expression levels was constructed. The combined model demonstrated improved discriminative performance compared with individual genes alone, as reflected by an increased AUC.

## 4. Discussion

Since its initial description by James Parkinson in 1817 [36], several mechanisms have been proposed to explain the pathogenesis of PD, yet its exact molecular pathophysiology remains inconclusive [37]. Moreover, although several omics studies have contributed to the knowledge of PD-associated genes/proteins and pathways [38], much of the complex architecture of PD is still unknown. In recent years, the m6A methylation modification—the most frequent internal RNA modification in eukaryotic coding and non-coding transcripts—has emerged as a pivotal component in the pathogenesis of neurodegenerative diseases such as PD [39,40]. However, these studies have focused mainly on the analysis of brain tissues from non-Latin American cohorts. Although peripheral tissues, such as whole blood, are not the primary target tissue of neurodegenerative diseases including PD, this is a practical tissue where medium–large-scale noninvasive sampling is feasible to evaluate the cellular damage in the brain and nervous system [40,41,42]. Furthermore, blood exhibits significant gene expression correlations with multiple central nervous system tissues, particularly for immune and inflammation-related pathways [43]. Therefore, peripheral blood transcriptomics will provide a minimally invasive window into systemic molecular alterations and immune-associated processes that contribute to or reflect disease progression associated with neurodegenerative disorders. Nevertheless, the relationship between peripheral blood transcriptomic alterations and molecular processes occurring in the central nervous system remains incompletely understood. Several studies have reported an altered expression of m6A regulatory factors in postmortem brain tissues and experimental models of PD, including changes affecting members of the *YTHDF* family and components of the m6A methyltransferase complex [20,39,40,44]. However, the extent to which peripheral expression patterns mirror transcriptional dynamics within neuronal tissues is still unclear. Therefore, the peripheral transcriptomic signatures identified in this study should be interpreted primarily as systemic molecular alterations associated with PD rather than direct proxies of neuronal epitranscriptomic regulation. Therefore, the present study sought to investigate m6A-associated transcriptional alterations in iPD using peripheral blood RNA-seq data from a Latin American cohort within the AMP-PD initiative.

The analysis of differential gene expression between early iPD patients and control subjects revealed a prominent gene downregulation pattern (97.47% and 2.53% of DEGs exhibiting downregulation and upregulation, respectively) in early iPD patients. Similar patterns have been previously reported in transcriptomic studies of blood and midbrain samples from early PD patients [44,45], suggesting that widespread transcriptional repression may represent a recurrent molecular feature of early disease stages. One possible interpretation is that this pattern reflects an increased contribution of post-transcriptional regulatory mechanisms in early iPD, potentially involving epitranscriptomic processes such as m6A-mediated RNA regulation. Nevertheless, we acknowledge that transcriptome-wide shifts toward downregulation could also be influenced by technical factors inherent to RNA-seq differential expression analyses. However, the use of standardized normalization procedures and covariate-adjusted statistical modeling in the present study reduces the likelihood that the observed pattern is solely attributable to technical bias. A relevant functional enriched category of the identified differentially expressed genes was the immune response. Although PD is not formally recognized as an autoimmune disease, emerging results suggest a significant role of immune dysregulation associated with an exacerbated pro-inflammatory response in the development of iPD [46,47]. Notably, m6A methylation is a crucial regulatory mechanism in pro-inflammatory responses, where it can enhance inflammation by promoting the expression of pro-inflammatory genes [48]. Our integrated approach, based on DEGs, WGCNA-hub genes and the human m6A-related genes database, revealed a total of twelve overlapping iPD m6A-related candidate genes (ten coding protein genes and two pseudogenes), with the majority (66.67%) being downregulated in early iPD patients. Six of the ten overlapped coding protein for iPD m6A-related genes candidates (*MAGI1*, *HTR4*, *NRCAM*, *PTPN14, PKHD1* and *KIR3DL1*) are key genes involved in recognized pathophysiology pathways strongly implicated in PD. However, given the modest sample size of the present study, the identification of these twelve overlapping genes should be interpreted cautiously, as some associations may arise by chance.

*MAGI1* encoded a scaffolding membrane-associated guanylate kinase homologue protein with a relevant role in cell–cell junctions, signaling, maintenance of cell polarity and cell-to-cell communication [49]. The depletion of *MAGI1* has been associated with an increase in the expression of *LATS* (Large Tumor Suppressor) 1 and 2 genes [50], which are key components of the Hippo signaling pathway. It should be noted that dysregulation of the Hippo signaling pathway contributes to PD by promoting oxidative stress, neuroinflammation and mitochondrial dysfunction, which are all key pathological features of PD [51,52]. Therefore, the transcriptional downregulation of *MAGI1* observed in peripheral blood may be associated with alterations in Hippo signaling-related pathways in early iPD. *HTR4* is a recognized member of the family of serotonin receptors, which are widely expressed in nearly all circulating immune cells [53,54]. Downregulation of serotonin receptors in blood is frequently associated with impaired platelet function and increased systemic inflammation [55]. Notably, functional abnormalities in platelets and inflammation are well-documented characteristics in PD patients [56,57]. *KIR3DL1*, the only upregulated overlapped iPD m6A-related gene observed in our results, is a well-recognized member of the family of killer cell immunoglobulin-like receptors (KIRs) normally expressed in T and natural killer cells and mainly associated with the suppression of natural killer cells activities, consequently preventing cell lysis [58]. A recent study has suggested that high blood *KIR3DL1* expression levels are associated with the reduction in the severity of several PD symptoms [59]. Based on these observations, we hypothesize that the upregulation of *KIR3DL1* identified in our study might represent a potential compensatory mechanism against early iPD progression, although further functional validation is required to confirm this role. *NRCAM* encodes a neuronal cell adhesion protein with expression in multiple tissues, which is required for normal cell–cell communications in brain and in the peripheral nervous system [60]. In vitro studies have revealed that an over-expression of *NRCAM* induces accelerated cell growth and migration while decreasing cell apoptosis activity [61]. In this context, the downregulation of *NRCAM* observed in our results is consistent with an increase in apoptosis activity, which has been suggested as the main mechanism associated with neuronal death in PD [62]. Furthermore, *PTPN14* is a recognized member of the protein tyrosine phosphatase family, which performs a pivotal role in the regulation of several cellular processes, including lymphangiogenesis [63]. The lymphatic brain system is crucial for removing macromolecular proteins (e.g., α-synuclein) from the brain, whose dysfunction has been well-documented in PD patients [64]. Accordingly, the transcriptional blood *PTPN14* downregulation observed in this study could reflect the dysregulation of the lymphatic brain system in early iPD patients. *PKHD1* encodes the fibrocystin protein, a protein found in epithelial cells of the kidneys and liver that plays a crucial role in the formation and maintenance of kidney and liver tubules [65]. Several studies have evaluated the effect of *PKHD1* depletion, showing that it increases cell apoptosis and kidney cyst development, primarily in the proximal tubules, thus affecting normal kidney function [65,66,67]. Given the emerging hypothesis proposing that impaired kidney function could increase PD risk [68], our findings regarding *PKHD1* expression lead us to speculate on a possible, yet uncharacterized, m6A-mediated regulatory link within the kidney–brain axis in PD pathogenesis. To further evaluate the relationship between the overlapped candidates iPD m6A-related genes and m6A RNA methylation regulatory factors (m6ARFs) a differential co-expression analysis was performed, showing three m6ARFs (*VIRMA, HNRNPA2B1* and *YTHDF3*) and two overlapped candidate iPD m6A-related genes (*NRCAM* and *PKHD1*) involved in significant transcriptional rewiring relationships. From a regulatory perspective, these interactions suggest that altered activity of specific m6A writers and readers may influence the epitranscriptomic marking of transcripts involved in cellular communication and survival pathways. In particular, *VIRMA* functions as a component of the m6A methyltransferase complex that guides methylation deposition on target transcripts, whereas the reader proteins *YTHDF3* and *HNRNPA2B1* participate in the recognition of m6A-modified RNA and can influence mRNA stability, translation efficiency, or RNA processing. Within this regulatory context, *NRCAM* encodes a neuronal cell adhesion molecule implicated in axonal guidance, synaptic organization, and neuronal connectivity—processes frequently disrupted in neurodegenerative disorders. Changes in m6A-associated regulation of *NRCAM* transcripts could therefore influence RNA stability or translation dynamics, although this possibility remains to be experimentally demonstrated. Similarly, *PKHD1* encodes fibrocystin, a protein involved in epithelial signaling and primary cilium function in renal tubular cells. Although its role in neurodegeneration remains poorly understood, emerging evidence suggests that renal dysfunction and systemic metabolic alterations may influence PD risk. In this context, altered m6A enrichment of *PKHD1* transcripts may reflect epitranscriptomic regulatory changes linked to systemic physiological processes, potentially consistent with the emerging hypothesis of a kidney–brain axis contributing to PD pathogenesis.

Notably, the detected regulator–target pairs exhibited consistent directionality of correlation changes between iPD and control groups. Rather than indicating widespread disruption of the m6A network, the restriction of differential co-expression to a limited subset of regulator–target interactions may reflect selective regulatory rewiring in early-stage PD. Network-based transcriptomic studies in PD have demonstrated that disease-associated perturbations frequently manifest as coordinated alterations within specific gene modules, rather than as global transcriptional collapse [69]. These findings support the concept that early PD may involve focal network-level dysregulation preceding large-scale transcriptomic disorganization. In this context, discrete m6A-associated regulatory changes may represent early systemic molecular signatures that parallel or contribute to PD-related molecular vulnerability. The MeRIP-qPCR results showed significant higher methylation levels of *NRCAM* and *PKHD1* in early iPD patients compared to control subjects, consistent with the differential co-expression patterns identified in the discovery dataset. This suggests that the m6A modifications are increased on the transcripts of these genes, which could potentially affect their lower expression and function. However, direct mechanistic effects on RNA stability or translation were not evaluated in this study. In addition, the evaluation of the transcriptional activity of the involved m6ARFs showed that *VIRMA* and *YTHDF3* were transcriptionally upregulated in iPD patients and that *HNRNPA2B1* was downregulated. Furthermore, ROC analyses were conducted to preliminarily assess the discriminative patterns of peripheral *YTHDF3*, *VIRMA*, and *HNRNPA2B1* expression. Each gene exhibited moderate classification performance, and the combined multivariable model showed a relative improvement compared with individual markers. Although these observations were consistent with the independent transcriptional validation analyses described above, the limited sample size warrants cautious interpretation and will require validation in larger independent cohorts before any conclusions regarding diagnostic utility can be drawn. *VIRMA* is a m6ARF writer that interacts with the catalytic domains of other m6ARF writers (e.g., *METTL3*, *METTL14* and *WTAP*) to guide them to specific RNA sectors for m6A methylation [70]. Recent brain studies have shown that the expression levels of *VIRMA* are altered in PD patients and murine PD models, where elevated expression of *VIRMA* has been reported; this contributes to the activation of neurodegeneration processes by suppressing the translation of the Parkin (PRKN) protein, which is essential for clearing damaged mitochondria [71,72]. In this study, *VIRMA* expression was increased in peripheral blood from early iPD patients. While this finding is directionally consistent with previous reports describing altered m6A machinery components in PD models, our data do not establish whether peripheral *VIRMA* expression reflects similar regulatory dynamics in neuronal tissue. Therefore, the observed upregulation of *VIRMA* in blood should be interpreted as a systemic m6A-associated transcriptional alteration rather than direct evidence of PRKN translational regulation or neurodegenerative mechanisms. Further mechanistic studies in disease-relevant neuronal models will be necessary to clarify whether *VIRMA*-mediated m6A modulation contributes to mitochondrial dysfunction in Parkinson’s disease. *YTHDF3* and *HNRNPA2B1* are two m6ARFs readers that regulate mRNA expression through of the recognition and combination of specific *HNRNPA2B1* and *YTHDF3* m6A motifs on mRNAs [68]. *YTHDF3* binds to m6A-modified YTH motifs on mRNAs in the cytoplasm, where it enhances translation or promotes mRNA decay [69]. Previous studies have reported complex PD transcriptional activity of *YTHDF3* depending on the brain region evaluated, being downregulated in the frontal cortex and cingulate gyrus and upregulated in the cerebellum [44]. In comparison, *HNRNPA2B1* binds to m6A-modified RRM 1/2 motifs on mRNA in the nucleus and cytoplasm and mainly participates in splicing processes and microRNA processing [70]. Interestingly, a recent study using a human tau transgenic fly model has suggested that *HNRNPA2B1* knockdown increases the toxic role of tau proteins [71]. Tau proteins are currently recognized as one of the earliest causes of neuronal damage and cell death in patients with PD [72].

Despite these findings, several limitations should be acknowledged. First, the modest sample size of the discovery cohort may increase the risk of false-positive associations in high-dimensional analyses such as WGCNA and differential co-expression, despite the analytical safeguards implemented to enhance network stability. Second, the study relied on peripheral blood transcriptomic data, which may not fully capture disease-relevant molecular processes occurring in the central nervous system. Third, most patients in the validation cohort were receiving dopaminergic therapy at the time of blood collection, which could potentially influence peripheral gene expression profiles. Because the limited cohort size precluded stratified analyses according to medication exposure, future studies including larger cohorts with detailed treatment metadata will be necessary to disentangle medication-related effects from disease-associated molecular signatures. Finally, experimental procedures were not conducted under blinded conditions. Taken together, these considerations indicate that the present findings should be interpreted as exploratory and will require validation in larger independent cohorts as well as mechanistic investigation in disease-relevant experimental systems.

## 5. Conclusions

In summary, this integrative transcriptomic analysis identifies m6A-associated differential expression and co-expression patterns in early iPD within a Latin American cohort. By combining network-based and differential co-expression approaches, we delineate candidate m6A regulator–target interactions that may contribute to systemic transcriptional signatures linked to immune signaling and neuronal-related pathways. These findings support the concept that selective m6A-associated regulatory rewiring may occur in early-stage disease and provide a framework for investigating epitranscriptomic contributions to PD pathophysiology. Given the historical underrepresentation of Latin American populations in large-scale transcriptomic initiatives, this study offers novel region-specific molecular insights that expand the current understanding of PD heterogeneity and warrant further validation in larger and ethnically diverse cohorts.

## Figures and Tables

**Table 1:** Significant m6A regulator–target differential co-expression links identified in early iPD. Correlations were calculated separately for control and iPD groups using Pearson correlation on VST-normalized expression data. Differential co-expression was assessed using Fisher’s Z-transformation with permutation-based testing (1000 permutations). *p*-values were adjusted using the Benjamini–Hochberg method (FDR). |Correlation change| denotes the absolute difference in correlation coefficients between groups (|Δr|).

Gene A	Gene B	Correlation Control	Correlation iPD	|Correlation Change|	*p*_Value	FDR
*NRCAM*	*VIRMA*	−0.7684	−0.1453	0.7231	0.0079	0.0143
*PKHD1*	*HNRNPA2B1*	−0.7201	−0.0331	0.7870	0.0062	0.0143
*NRCAM*	*YTHDF3*	−0.7756	−0.2130	0.7627	0.02130	0.0317

## Data Availability

Data used in the preparation of this work were obtained from the Accelerating Medicine Partnership^®^ (AMP^®^) Parkinson’s Disease (AMP PD) Knowledge Platform. This data availability on TERRA (https://terra.bio) server from The AMP^®^ PD program is a public–private partnership managed by the Foundation for the National Institutes of Health and funded by the National Institute of Neurological Disorders and Stroke (NINDS) in partnership with the Aligning Science Across Parkinson’s (ASAP) initiative; Celgene Corporation, a subsidiary of Bristol-Myers Squibb Company; GlaxoSmithKline plc (GSK); The Michael J. Fox Foundation for Parkinson’s Research; AbbVie Inc.; Pfizer Inc.; Sanofi US Services Inc.; and Verily Life Sciences. For up-to-date information on the study, visit https://www.amp-pd.org.

## Supplementary Materials

The following supporting information can be downloaded at https://www.mdpi.com/article/10.3390/life16040592/s1. Supplementary Table S1: Metadata of AMP-PD samples analyzed in the present study (cohort discovery); Supplementary Table S2: Details of m6A RNA methylation regulatory factors included in this study; Supplementary Table S3: Metadata of iPD Latin American patients included in this study (cohort validation); Supplementary Table S4: Sequences of gene-specific primers to RT-qPCR and meRIP-qPCR; Supplementary Table S5: List of DEG among control and early iPD; Supplementary Table S6: Enriched GO terms of DEG among controls and early iPD patients; Supplementary Table S7: Enriched KEGG pathways of DEG among controls and early iPD patients; Supplementary Table S8: Gene annotation and functional description of the overlapping genes detected in this study; Supplementary Figure S1: Study workflow for the identification and validation of m6A regulators in early iPD; Supplementary Figure S2: Robust outlier detection and sensitivity analysis of the study cohort; Supplementary Figure S3: Surrogate variable analysis and assessment of potential confounding effects in peripheral blood RNA-seq data; Supplementary Figure S4: Protein–protein interaction network of 12 overlapping iPD m6A-related genes.
