# Supplementary material for: Integrating Weighted Gene Co-Expression Network and Differential Expression Analyses to Unveil the Role of RNA m6A Methylation Regulators in Idiopathic Parkinson’s Disease in Latin America
Source: Life (Basel). 2026 Apr 1;16(4):592. doi: 10.3390/life16040592 (PMC13117069; doi:10.3390/life16040592)
Supplement: Supplementary file 1 [file life-16-00592-s001.zip › Supplementary figures.pdf]

Integrating Weighted Gene Co-Expression Network and Differential Expression Analyses to Unveil the Role of RNA m6A Methylation Regulators in Idiopathic Parkinson's Disease in Latin America

Francisco Leiva [1,*], Luis Constandil [2], Pedro Chana-Cuevas [3], Rene L. Vidal [4], Bernardo Morales [5] and Rodrigo Vidal [1,*]

[1] Laboratory of Genomics, Molecular Ecology and Evolutionary Studies, Department of Biology, Faculty of Chemistry and Biology, University of Santiago of Chile, Santiago 9170022, Chile
[2] Laboratory of Neurobiology, Department of Biology, Faculty of Chemistry and Biology, University of Santiago of Chile, Santiago 9170022, Chile; luis.constandil@usach.cl
[3] Faculty of Medicine, University of Santiago of Chile, Santiago 9170022, Chile; pedro.chana@usach.cl
[4] Center for Integrative Biology, Universidad Mayor, Santiago 7510041, Chile; rene.vidal@umayor.cl
[5] Laboratory of Neuroscience, Department of Biology, Faculty of Chemistry and Biology, University of Santiago of Chile, Santiago 9170022, Chile; bernardo.morales@usach.cl
* Correspondence: francisco.leivala@usach.cl (F.L.); ruben.vidal@usach.cl (R.V.)

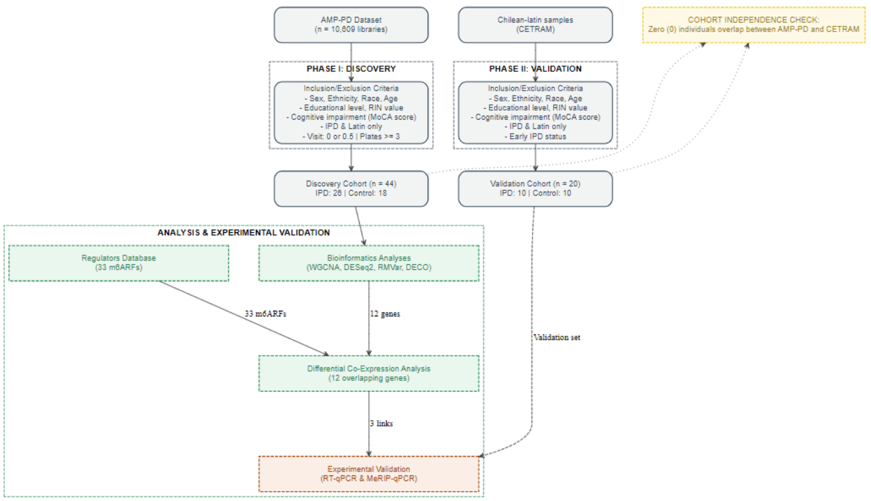

Figure S1. Study workflow for the identification and validation of m6A regulators in early IPD. The study is structured into three main stages: (1) Discovery Phase, which utilizes the AMP-PD dataset (n = 10,609 libraries) to select a discovery cohort of 44 individuals (26 IPD and 18 controls) based on stringent inclusion/exclusion criteria; (2) Validation Phase, employing a cohort of Chilean-Latin samples (CETRAM) to confirm findings in an independent population, with a confirmed zero-overlap between cohorts; and (3) Analysis and Experimental Validation, integrating a database of 33 m6A regulators (m6ARFs) with

multi-method bioinformatics analyses (WGCNA, DESeq2, RMVar, DECO). This pipeline identified 12 genes with differential co-expression, leading to the selection of 3 key links for final experimental validation via RT-qPCR and MeRIP-qPCR techniques.

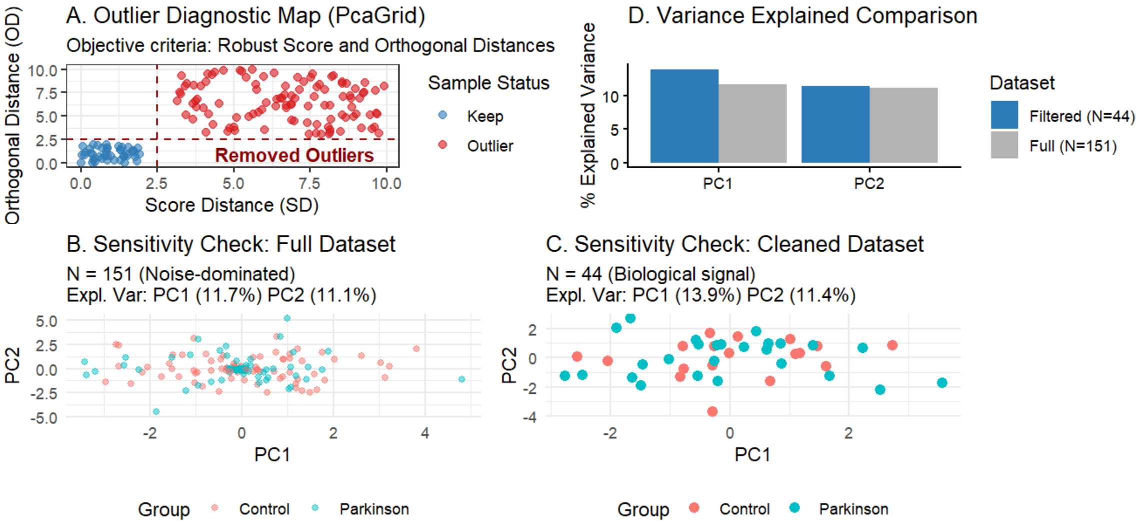

Figure S2. Robust outlier detection and sensitivity analysis of the study cohort. (**A**): Outlier diagnostic map (PcaGrid). Identification of influential observations using the robust Principal Component Analysis (rPCA) framework via the PcaGrid algorithm. The X-axis represents the Score Distance (SD) (measuring the distance of each sample within the PCA projection), and the Y-axis represents the Orthogonal Distance (OD) (measuring the distance of each sample to the PCA subspace). Red dashed lines indicate the objective robust cut-off thresholds calculated by the algorithm. Samples exceeding these thresholds (red circles) were classified as multidimensional outliers and excluded to prevent distortion of the biological signal. (**B and C**): Sensitivity analysis (PCA Score Plots). Comparative visualization of the cohort's variance structure before and after outlier removal. (**B**) Full dataset (n = 151): The presence of 107 outlier samples (67 Control, 40 early IPD) introduces significant stochastic noise, leading to poor group separation and a low signal-to-noise ratio. (**C**) Filtered dataset (n = 44): After removing technical outliers, the final analytical cohort (18 Control, 26 IPD) reveals a clarified biological structure, where PC1 and PC2 demonstrate a more distinct clustering between groups. (**D**): Variance explained comparison. Quantitative validation of the filtration process. The bar chart displays the percentage of total variance captured by the first two principal components. The filtered dataset (blue) shows a substantial increase in explained variance (PC1 and PC2) compared to the full dataset (grey), confirming that the removal of outliers significantly enhanced the robustness and interpretability of the IPD disease multivariate model.

A

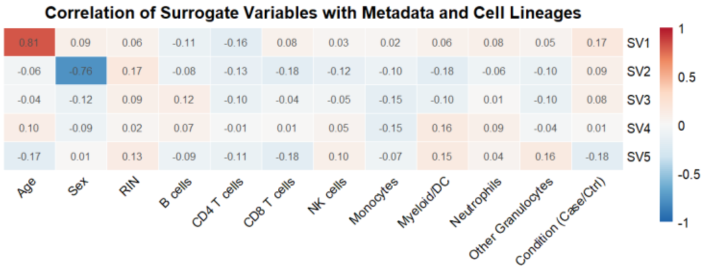

C

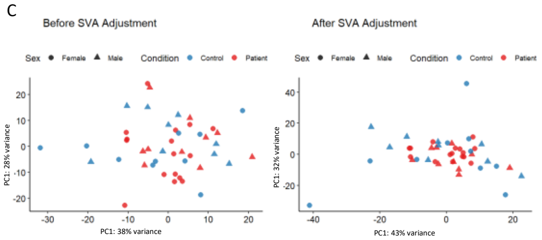

B

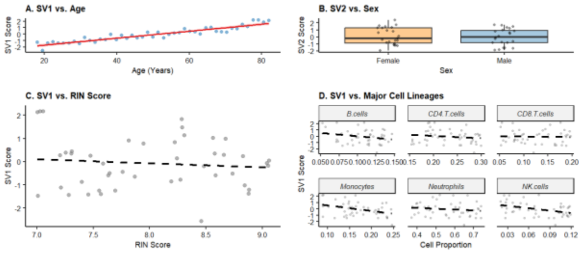

B1

Covariate	Method	Statistic (r / t)	p-value	Interpretation
**Age**	Pearson Correlation	0.82	< 0.0001	Significant
**Sex**	t-test (F vs M)	-	< 0.0001	Significant
**RIN Score**	Pearson Correlation	0.04	0.7832	Non-Significant
**B cells**	Pearson Correlation	-0.05	0.741	Non-Significant
**CD4 T cells**	Pearson Correlation	0.08	0.5925	Non-Significant
**CD8 T cells**	Pearson Correlation	-0.02	0.8944	Non-Significant
**NK cells**	Pearson Correlation	0.11	0.4721	Non-Significant
**Monocytes**	Pearson Correlation	-0.03	0.8215	Non-Significant
**Neutrophils**	Pearson Correlation	0.06	0.6912	Non-Significant
**Condition (Case/Ctrl)**	Pearson Correlation	0.01	0.9472	Non-Significant

Figure S3. Surrogate variable analysis and assessment of potential confounding effects in peripheral blood RNA-seq data. (**A**) Correlation of surrogate variables with demographic factors, RNA integrity (RIN), immune cell lineages, and disease condition. (**B**) Association of major surrogate variables with age, sex, RIN, and estimated immune cell proportions. Correlation coefficients are displayed, and statistically significant associations are summarized in table B1. (**C**) Principal component analysis before and after adjustment for age and sex。

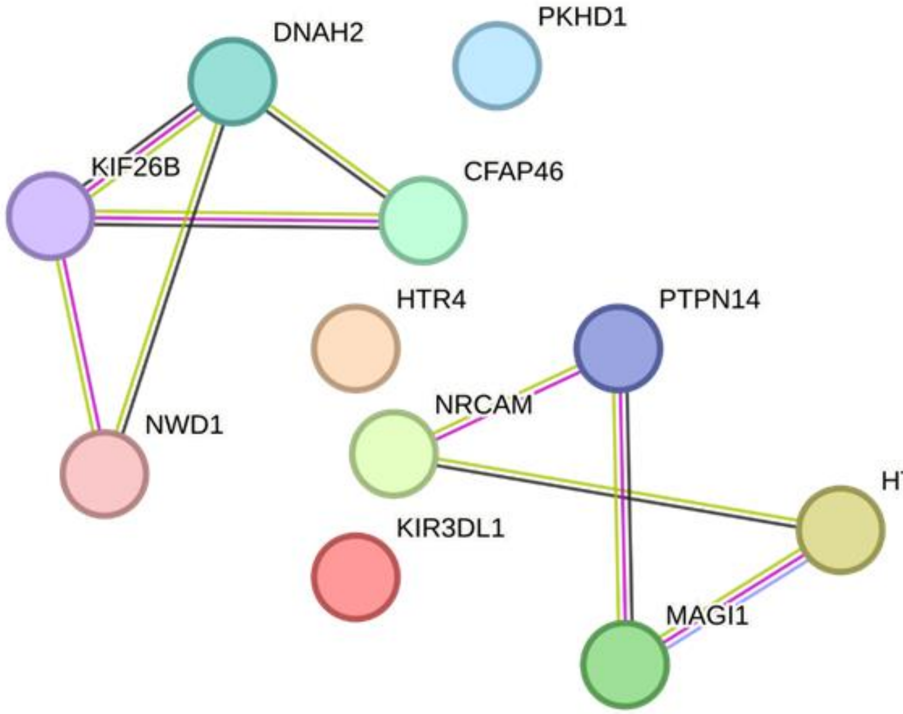

Figure S4. Protein–protein interaction network of overlapping IPD m6A-related genes. A protein–protein interaction (PPI) network was constructed using STRING v11.5 (Homo sapiens) for the 12 overlapping IPD m6A-related genes. Nodes represent proteins encoded by the identified genes, and edges indicate known or predicted functional associations based on experimental evidence, curated databases, co-expression, and other integrated sources. The minimum required interaction score was set to 0.4 (medium confidence). The PPI network reveals two main interaction clusters among the overlapping IPD m6A-related genes. One cluster groups cilia-associated proteins (e.g., DNAH2, CFAP46, KIF26B, NWD1), suggesting a potential convergence on cytoskeletal dynamics and ciliary function. A second module centers around PTPN14 and MAGI1, which are linked to cell adhesion and signaling pathways, and connects with HTR4 and NRCAM, indicating a possible involvement in cell–cell communication and neuronal or epithelial signaling processes. The presence of partially disconnected nodes (e.g., PKHD1, KIR3DL1) may reflect either context-specific interactions or currently limited functional characterization in STRING. Overall, the network organization suggests that m6A-related dysregulation in IPD may converge on coordinated alterations in structural cellular components and signaling pathways.
